# Case of Dysodontophysis

**Published:** 1805-06-01

**Authors:** 


### *To the Editors of the Medical and Physical Journal.*

GENTLEMEN,

MR. R. O. Millett, jun. of Hayle, in Cornwall, has the thanks of Academicus for his letter, in p. 51 of your 12th volume. It seems that, whether, according to Mr. Cuming, (p. 518 of the 11th volume) cold was the remote cause of the child's illness, or, according to Mr. M. indigested sordes and accumulation of slime occasioned it, an emetic, in the first instance, is the practice of both: and had no confidential practitioner resided within several miles of the child, an emetic would, most probably, in the first instance, have been administered to him by a relative resident in the house with him. But the apothecary justifies the different treatment pursued by himself, in the following letter to the father of the child:

"SIR, *June* 21, 1804.

"In answer to yours of yesterday, I beg to say, the leading circumstances of your little boy's illness are perfectly fresh in my mind; and that the practice pursued by me on the occasion was such, as to leave not the smallest reflection, or require the least *justification* whatever. That cold * was the remote cause of the disease, that the capillaries were in consequence contracted, (and that perspiration, which originally escaped through them, thrown into the circulation) no one had the least doubt; but that

 an

---

* Then why was it asserted to the father and mother, that it was *bile;* and that if it had been *cold*, it would have been indicated by *cough?*

an emetic was necessary, or even proper, at the time I first saw him, I most decidedly object to. First, because no sickness or nausea, or indeed any symptom whatever, appeared to call for it; and, 2dly, because a diarrhœa of green bilious matter having commenced, it was just and right to conclude, Nature had pointed out that the path to be pursued; and that the best and only means of seconding her efforts, was by giving a laxative, of which calomel had the preference, and which practice the candid and liberal Mr. Cuming does not object to. I must again insist on the impropriety of giving an emetic, which, by its revulsive and counteracting effects, would have had the worst of consequences; whilst the plan pursued was both judicious and rational, and from which the most beneficial effects were expected. Again, if an emetic was absolutely requisite, why did not Dr. A. order it when called in, at which time the condition of the child was not† materially altered? Why, for the best of all possible reasons, because it was not necessary, and its exhibition, to say no worse of it, would have been bad practice. The very indecent, ungentlemanlike, and unhandsome observations of Mr. C. upon the case in question, require some notice; but which I shall defer till a more favourable opportunity.

"I am, &c.

"B. D."

"P. S. The saline mixture and blister were neither of them ordered with a view of relieving sickness (none existing) but with the object of promoting and causing a gentle diaphoresis, thereby counteracting the spasm upon the extreme vessels."

After this defence, the parties to whom it is made have read, in a book styled *The Modern Practice of Physic*, among the disorders of children, as follows: "Teething children, about the time of cutting their teeth, slaver much, and have generally a looseness. When the teething is difficult, especially when the dog-teeth begin to make their way through the gums, the child has *startings in his sleep*, tumours of the gums, *inquietude, watchings, gripes*, GREEN STOOLS, the thrush, *fever, difficult breathing*, and *convulsions*."

The child, whose case is in vol. 11, p. 461, of the Medical and Physical Journal, had all the symptoms above marked in

† The condition of the child was altered to great debility and emaciation and the physician, when called in, declared it a lost case.

in *italics,* and particularly *green stools,* which Mr. B. D. calls "*a diarrhœa of green bilious matter*" The child was also then cutting his last four teeth, at the two extremities of each jaw, having the intermediate sixteen perfectly cut; all of which had been cut with difficulty, as Mr. B. D. the family apothecary, had the means of knowing. *Unless,* therefore, it is wrong to ascribe *green stools* to *teething,* and they are *always* indications of *bile,* Mr. B. D. will have to reflect, whether *a blister* (on the breast of a child two years old) *six inches or more by three,* and *calomel,* would have *the best* effects in difficult dentition. *If* they would *not,* then it is left to him to reflect, *why* he did not, by proper interrogatories, *first* learn the state of the teeth, before he ascribed the green stools solely to bile, and treated the child accordingly.

